# Foot-and-Mouth Disease Virus-Associated Abortion and Vertical Transmission following Acute Infection in Cattle under Natural Conditions

**DOI:** 10.1371/journal.pone.0167163

**Published:** 2016-12-15

**Authors:** Rajeev Ranjan, Jitendra K. Biswal, Saravanan Subramaniam, Karam Pal Singh, Carolina Stenfeldt, Luis L. Rodriguez, Bramhadev Pattnaik, Jonathan Arzt

**Affiliations:** 1 ICAR- Directorate of Foot and Mouth Disease, Mukteshwar, Nainital, Uttarakhand, India; 2 CADRAD, IVRI Izatnagar, Bareilly, Uttar Pradesh, India; 3 Foreign Animal Disease Research Unit, USDA-ARS, Plum Island Animal Disease Center, Greenport, New York, United States of America; 4 Oak Ridge Institute for Science and Education, PIADC Research Participation Program, Oak Ridge, Tennessee, United States of America; University of Minnesota College of Veterinary Medicine, UNITED STATES

## Abstract

Foot-and-mouth disease (FMD) is a highly contagious and economically important viral disease of cloven-hoofed animals, including domestic and wild host species. During recent FMD outbreaks in India, spontaneous abortions were reported amongst FMD-affected and asymptomatic cows. The current study was an opportunistic investigation of these naturally occurring bovine abortions to assess causality of abortion and vertical transmission of FMDV from infected cows to fetuses. For this purpose, fetal tissue samples of eight abortuses (heart, liver, kidney, spleen, palatine tonsil, umbilical cord, soft palate, tongue, lungs, and submandibular lymph node) were collected and screened by various detection methods, including viral genome detection, virus isolation, and immunomicroscopy. Amongst these cases, gross pathological changes were observed in 3 abortuses. Gross pathological findings included blood-tinged peritoneal and pleural effusions and myocarditis. Hearts of infected calves had mild to moderate degeneration and necrosis of the myocardium with moderate infiltration by mixed inflammatory cells. Localization of FMDV antigen was demonstrated in lungs and soft palate by immunomicroscopy. FMDV serotype O viral genome was recovered from 7 of 8 cases. Infectious FMDV serotype O was rescued by chemical transfection of the total RNA extracted from three soft palate samples and was sequenced to confirm 100% identity of the VP1 (capsid) coding region with isolates collected from infected cattle during the acute phase of infection. Based upon these findings, it may be concluded that FMDV-associated abortion occurred among the infected pregnant cows included within this study and FMDV was subsequently transmitted vertically to fetuses. This is the first documentation of FMDV-associated abortions in cattle.

## Introduction

Foot-and-mouth disease (FMD) is a highly contagious and economically important viral disease of domestic cloven-hoofed animals including cattle, goats, sheep, pigs and more than 70 wild species [1, 2]. It is a major barrier to worldwide trade in animals and animal products. The disease is caused by FMD virus (FMDV), genus *Aphthovirus*, family *Picornaviridae* [2, 3]. The clinical phase of FMD is characterised by pyrexia, lameness and vesicular lesions of the oral cavity, feet, snouts of pigs, and teats of dairy cows [4, 5]. FMDV exists in seven serologically and immunologically distinct serotypes (O, A, C, Asia 1, SAT-1, -2, -3) [6], which are further sub-divided into multiple topotypes/genotypes based on phylogenetic properties [7, 8]. Although FMD-associated mortality is low in adult animals, high mortality is sometimes observed among young animals and is often attributed to acute myocarditis [4, 9, 10].

FMD is highly transmissible during the clinical phase of infection [11, 12]. The common mechanism of spread of the disease is by direct contact, and may occur by mechanical transfer of virus from infected to susceptible animals through damaged skin or intact mucosae or by deposit of droplets or droplet nuclei (aerosols) in the respiratory tract of susceptible animals [13–15].

The O/ME-SA/Ind2001 lineage of FMDV has intermittently dominated circulating FMDVs in India since it was first reported in 2001 [16]. Between June 2013 and January 2014, the sub-lineage FMDV-O/ME-SA/Ind2001d caused extensive outbreaks of FMD in India, particularly in the southern region of the country [17]. During that period, this lineage was also detected in field outbreaks in the northern Indian state of Uttarakhand and in neighbouring countries including Bhutan in 2013 [18], Nepal and Sri Lanka in 2014 [19]. FMDV-Ind2001d has also spread to the Middle East including Saudi Arabia in 2013 [18] and Bahrain in 2015 [20]. The lineage was also reported from North African countries including Libya in 2013 [21], Algeria in 2014, and Tunisia in 2014 [19].

Vertical transmission is defined as an ‘‘infection that is transferred from dam to embryo, fetus, or new-born before, during, or shortly after parturition”[22]. Vertical transmission of various DNA and RNA viruses has been documented in animals [23, 24]. Similarly, transplacental transmission of viruses have been reported by several workers [25–27]. Specifically, transplacental transmission of blue tongue virus serotype-8 was demonstrated in goats with infectious virus recovered from the caprine fetuses. In cattle, transplacental transmission has previously been demonstrated for bovine immunodeficiency virus (BIV) by Meas *et* al. [28] and in bovine viral diarrhoea virus (BVDV) by Trautwein *et al*. [29].

There has been limited description and investigation of vertical transmission of FMDV. Early reports suggested vertical transmission by describing FMD-associated abortions in sheep [30, 31]. Similarly, large numbers of abortions were documented to occur within 24 h of the onset of lameness in sheep flocks during the 2001 outbreak in the UK [32]. Furthermore, it was subsequently demonstrated that the same FMDV strain that caused the UK 2001 outbreak, was able to cross the placenta and cause fetal death in ewes under experimental conditions [33, 34]. In an expert opinion survey conducted to assess the financial impact of FMD in Turkey, it was reported that the impact of FMD on abortion rate in exotic cattle (Holstein) and sheep were almost two times higher than in local breeds of cattle and sheep as well as in goats [35]. However, there is no confirmed report demonstrating fetal infection or abortion caused by FMDV under natural (field) conditions in any species. In the present study, we provide the first evidence of naturally occurring abortion and vertical transmission of FMDV in cattle under field outbreak conditions.

## Materials and Methods

### Permissions and ethics

The work described herein was performed by federal staff of the Directorate of Foot-and-Mouth Disease, Indian Council of Agricultural Research, Ministry of Agriculture, Government of India. The work occurred and the animals were maintained within facilities that were owned, maintained, or overseen by this division of the federal government; thus, no permits or approvals were required. Two study sites in India were in Mukteshwar, Uttarakhand State and Bhilai, Chhattisgarh State. All cases described herein occurred spontaneously in domestic cattle with no experimentation, inoculation, or treatment of live animals. No animals were euthanized for the purpose of this study. Sample collection was performed as part of routine field outbreak investigations; samples were subsequently compiled for the sake of the current investigation.

### History and animals

The cases described herein occurred associated with field outbreaks of FMD in dairy cattle herds in Mukteshwar, Uttarakhand State (Oct. 2013) and Bhilai, Chhattisgarh State (Dec 2013) in India. Cattle were mixed breed as are commonly maintained in India for dairy production containing variable proportion Holstein Friesian lineage and native Bos indicus. In both herds, all cows were vaccinated regularly (twice yearly in Mukteshwar; 4 times yearly in Bhilai) with a commercially available inactivated FMDV-trivalent vaccine containing serotypes A, O, and Asia1. The Mukteshwar farm was a small facility operated by the Indian Council of Agricultural Research. At the time of the outbreak, this farm housed 40 mature dairy cows amongst which 25 new cases of FMD occurred during the 10 day period of the outbreak (incidence = 25/40; 62.5%). The Bhilai farm was a large commercial dairy operation at which 222 cases of FMD were detected amongst 1836 adult dairy cows over the course of 35 days (incidence = 222/1836; 12.1%). Clinical signs exhibited by cattle consisted of variable combinations of vesicular and erosive stomatitis, interdigital vesicles and erosions, feed refusal, lameness, and ptyalism. All clinically observed FMD infections were confirmed diagnostically by viral genome detection (GD) methods including RT-LAMP and multiplex RT-PCR (mRT-PCR). During and after the clinical FMD outbreak period, abortions were reported from clinically affected and asymptomatic cows. Based upon logistical availability, eight abortuses from the affected farms were presented for examination and sampling. Necropsies and sample collections of all calves were conducted by a veterinary pathologist.

### Sample collection

In order to investigate vertical/transplacental transmission of FMDV from infected cows to their fetuses, 10 tissue samples were collected from the aborted fetuses at necropsy examination. Tissues collected were: heart, liver, kidney, spleen, palatine tonsil, umbilical cord, soft palate, tongue, lungs, and submandibular lymph node. Tissues were examined by conventional pathology, viral genome detection tests (GD) and virus isolation (VI). At necropsy examination, duplicate tissue samples were collected and stored in 50% (v/v) buffered glycerin for VI and in 10% neutral buffered formalin for histopathology. Tissue samples for VI and serotype-differentiating antigen ELISA were processed as 10% emulsion of homogenised suspension in PBS, the lysates were centrifuged at 3000 × g for 15 min and the supernatants were stored at −80°C until analysis. These suspensions were used as antigen in serotype-determination ELISA carried out as previously described [36].

### RNA extraction and cDNA synthesis

The tissue samples in 50% glycerol phosphate buffer were used to prepare a 10% suspension in phosphate-buffered saline (pH: 7.4). Viral RNA was extracted from this suspension using QIAamp viral RNA mini kit (QIAGEN, Hilden, Germany) as per the manufacturer’s instruction. The extracted RNA was further purified and treated with DNaseI using RNeasy kit (QIAGEN, Hilden, Germany) and was quantified by NanoDrop^™^ 1000 spectrophotometer considering the OD260/OD280 ratio, which was 1.8–2.0 for all the samples. Total RNA preparations were stored at—80°C till further use. The cDNA synthesis was performed with Thermoscript ^™^ Reverse Transcriptase (Invitrogen, USA) enzyme and FMDV specific reverse primer, NK61 [37] at 55°C for 2 h.

### Viral nucleic acid detection

Total RNA extracted was used for detection of FMDV RNA by the RT-LAMP assay [38]. For further confirmation of FMDV serotype, multiplex -PCR was carried out as described earlier [39] using the cDNA prepared as mentioned above. The m-PCR mix included, three serotype specific forward primers namely DHP13, DHP15 and DHP9 against O, A and Asia1, respectively and FMDV specific reverse primer NK61, Serotypes were differentiated based on amplicon size (249, 376, 537 bp specific for serotype O, A and Asia1 respectively). Both m-PCR and RT-LAMP amplified products were resolved on 2% agarose gel electrophoresis and visualized by ethidium bromide staining.

### Virus isolation, sequencing, and phylogenetics

Rescue of infectious FMDV was performed by chemical transfection of the extracted total RNA from each tissue collected on BHK-21 cells as previously described [40]. In brief, 1 μg of extracted RNA and 2 μl lipofectamine 2000 (Invitrogen, Carls-bad, USA) diluted in OPTI-MEM^®^I (Gibco, Life Technologies, NY, USA) were mixed and kept at room temperature for 20 min. These mixtures were transferred to monolayer BHK-21cells in 24-well plates overlaid with 200 μl of GMEM (Thermo Fisher Scientific, Waltham, MA USA) and incubated for 4 h. After 4 h of incubation, 700 μl of GMEM was added and again incubated at 37°C for 48 h. Then the whole contents in the wells were harvested and stored at −80°C. 200 μl of harvest aliquot was subjected to further passage in BHK-21cells in order to amplify the virus rescued by transfection for further use.

For phylogenetic comparisons, PCR amplification of VP1 coding regions of FMDV were performed using the primer combination of ARS4 [41] and NK61 in combination with Fermentas Pfu DNA polymerase (Thermo Fisher Scientific, Waltham, MA USA) as previously described [16]. The PCR products were purified using QIA quick Gel Extraction Kit (QIAGEN, Hilden, Germany). Purified VP1 amplicons were sequenced on an ABI 3130 automated DNA sequencer (Applied Biosystems, CA, USA) with the Big Dye Terminator v3.1 (Thermo Fisher Scientific, Waltham, MA USA) cycle sequencing kit. Sequences were aligned using clustal W algorithm and genetic analysis was performed using MEGA 6.06 software [42].

### Histological examinations

Tissue samples fixed in 10% neutral buffer formalin were processed by routine methods prior to embedding in paraffin and microtomy at 5μm sections. Routine histopathological examination (haematoxylin and eosin stain; H&E), as well as FMDV antigen detection by immunohistochemistry (IHC) and indirect fluorescence assay (IFA) were carried in different tissues as described by Arzt *et al*.[43]. Antigens were retrieved by microwave heating for 20 min in tris EDTA (pH-9.2). FMDV-infected bovine tissues, previously confirmed with GD methods, were used as positive control and GD-negative tissues from an apparently healthy cow collected from slaughter house were used as negative control. Additional negative controls included duplicates of each experimental section incubated with the primary antibody substituted with a species- and isotype-matched antibody derived from normal sera for polyclonal antibodies. Primary and secondary antibodies were validated and optimized previously by serial dilution using known positive FMDV infected tongue vesicle sections. FMDV antigen was detected by use of FMDV serotype O rabbit polyclonal antibody (dilution 1: 2000 in blocking buffer) as primary antibody [44]. The secondary antibody detection system was goat anti rabbit IgG HRP and goat anti rabbit IgG Rodamine for IHC and IFA, respectively as per manufacture instruction (Santa Cruz Biotechnology).

## Results

### Clinical characteristics of the outbreak

All cases described herein occurred spontaneously in well-organized professional dairy farms in north and central India in 2013–2014. All cows had been previously vaccinated with a commercially available inactivated FMDV-trivalent vaccine containing serotypes A, O, and Asia1. Adult cows that had clinical signs of FMD had vesicular and erosive stomatitis, interdigital vesicles and erosions, feed refusal with profuse salivation, lameness, and were subsequently diagnosed as FMDV-infected by GD (m-PCR and RT-LAMP) methods. During and after the onset of FMD in the herds, abortions and death of neonatal calves were reported amongst clinically affected and asymptomatic cows. In the current report, 8 cases of naturally occurring abortion were available to investigate causality of abortion and vertical transmission of FMDV from infected dams (Table 1).

**Table 1:** Clinical status of FMDV infected cows and post-mortem findings in aborted fetuses and dead calves.

Dam ID	FMD status	Gestational age when infected	Abortion/ Parturition day (dpm*)	Gross lesions in fetus
454	Clinical FMD	8 months 20 days	4	Bicavitary effusions, Myocarditis,
571	Clinical FMD	8 months 25 days	10**	Bicavitary effusions, Myocarditis,
483	Asymptomatic	4 months^#^	42^#^	None
271	Asymptomatic	6 months 15 days^#^	45^#^	None
107	Clinical FMD	5 months 12 days	46	None
450	Clinical FMD	1 month	150	Bicavitary effusions
372	Clinical FMD	3 months 10 days	150	None
562	Clinical FMD	3 months	160	None

*dpm = days post onset of clinical manifestation of FMD

**Calf died five days after birth

^#^day of infection based upon onset of FMD in the herd; dpm calculated as elapsed time between abortion and onset of FMD in the herd

### Macroscopic and microscopic lesions

**Fig 1:**
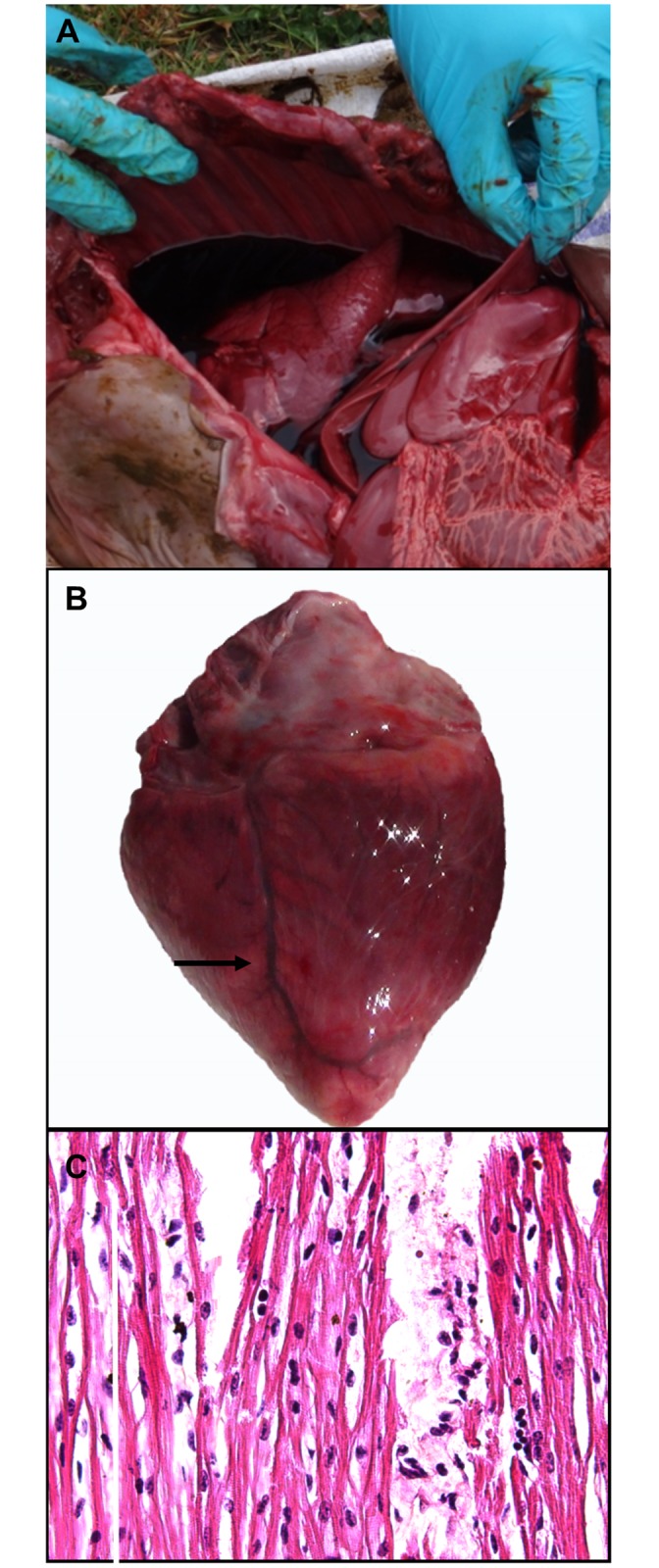
Gross and histopathological findings of FMDV-associated myocarditis in still-borne calf. **(A)** Sanguineous bicavitary effusions in still-borne calf from Dam ID#454. **(B)** Focal myocarditis seen as area of pallor (arrow) on epicardial surface of heart from aborted calf shown in panel (A). **(C)** Mixed mononuclear infiltrate and interstitial edema separating myocardial fibers in histological specimen from heart. Hypereosinophilia and loss of striations indicates myofiber degeneration and necrosis. Hematoxylin and Eosin stain. 40x magnification.

Gross pathological changes were present in three out of 8 calves including myocarditis (2/8) and blood-tinged peritoneal and pleural effusions (3/8). (Table 1; Fig 1A). Mild to moderate multifocal pallor of the myocardium was detected in hearts of two affected calves and was interpreted as presumptive myocarditis (Fig 1B). Petechial haemorrhage and congestion of the splenic capsule were present in one aborted fetus. No significant gross changes were observed in liver, kidney, palatine tonsil, umbilical cord, soft palate, tongue, brain, or sub-mandibular lymph node in any of the examined cases.

Microscopic analysis of grossly affected hearts confirmed the diagnosis of myocarditis with detection of moderate infiltration of inflammatory cells, consisting mainly of lymphocytes dissecting cardiac muscle bundles in affected myocardium (Fig 1C). Additionally, the lungs of two infected calves (Dam ID#s 454 and 571) had mild acute inflammation including interstitial and alveolar infiltrates of few neutrophils and modestly increased alveolar macrophages. Two fetuses had renal changes including mild infiltration of neutrophils within glomeruli and tubular vacuolation (not shown).

### Virus genome detection and chemical transfection from tissues

All 10 tissues collected at necropsy from each abortion case were subjected to chemical transfection and FMDV genome detection by RT-LAMP and m-PCR (Table 2). FMDV serotype O specific viral genome was detected by m-PCR from 7/8 cases (Fig 1) in a total of 27/80 tissues (Table 2). The highest prevalence of detection occurred in soft palate (7/8) and palatine tonsil (4/8). Additionally, viral RNA was detected in the submandibular lymph node (2/8), liver (1/8), kidney (2/8), and lungs (2/8). Infectious FMDV was rescued by transfection of BHK21 from only three out of eight cases and exclusively from the soft palate (two fetuses aborted from clinically affected and one fetus aborted from asymptomatic cow).

**Table 2:** FMD detection in tissues collected form aborted fetuses and dead calves.

Dam ID	454			571			483			271			107			450			372			562		
	A	B	C	A	B	C	A	B	C	A	B	C	A	B	C	A	B	C	A	B	C	A	B	C
Heart	-	**+**	-	-	**+**	-	-	-	-	-	**+**	-	-	-	-	-	-	-	-	-	-	-	-	-
Liver	-	**+**	**+**	-	-	-	-	-	-	-	-	-	-	-	-	-	-	-	-	-	-	-	-	-
Kidney	-	**+**	-	-	-	-	-	-	-	-	**+**	-	-	-	-	-	-	-	-	-	-	-	-	-
Spleen	-	**+**	-	-	**+**	-	-	-	-	-	**+**	-	-	-	-	-	-	-	-	-	-	-	-	-
Palatine tonsil	-	**+**	-	-	**+**	-	-	-	-	-	**+**	-	-	**+**	-	-	**+**	-	-		-	-		-
Umbilical cord	-	**+**	-	-	**+**	-	-	-	-	-	-	-	-	-	-	-	-	-	-	-	-	-	-	-
Soft palate	**+**	**+**	**+**	**+**	**+**	**+**	-	-	-	**+**	**+**	-	-	**+**	-	-	**+**	-	-	**+**	-	-	**+**	-
Tongue	-	**-**	-	-	-	-	-	-	-	-	-	-	-	-	-	-	-	-	-	-	-	-	-	-
Lungs	-	**+**	**+**	-	**+**	**+**	-	-	-	-	-	-	-	-	-	-	-	-	-	-	-	-	-	-
Sub-mandibular LN	-	**+**	-	-	**+**	-	-	-	-	-	-	-	-	-	-	-	-	-	-	-	-	-	-	-

A. FMD virus rescued by transfection, B. Viral Genome detection, C. Immunomicroscopy (immunohistochemistry and immunofluorescence)

Overall, there was a trend of decreasing tissue-level prevalence of FMDV RNA with progressive elapsed time from the period of clinical FMD of the dam. In the single case in which abortion occurred after 4 day post clinical manifestation (dpm) (dam ID#454), 9 out of 10 different types of tissues were found to contain viral genome(Table 2). In the case of the abortion of dam ID 571 at 10dpm, 7 tissues contained FMDV RNA. Interestingly, 5 tissues were RNA-positive in the abortus of a dam (ID# 271) who never had any clinical signs of FMD but was seropositive for FMDV non-structural protein 3AB3.

### Viral sequence analysis

**Fig 2:**
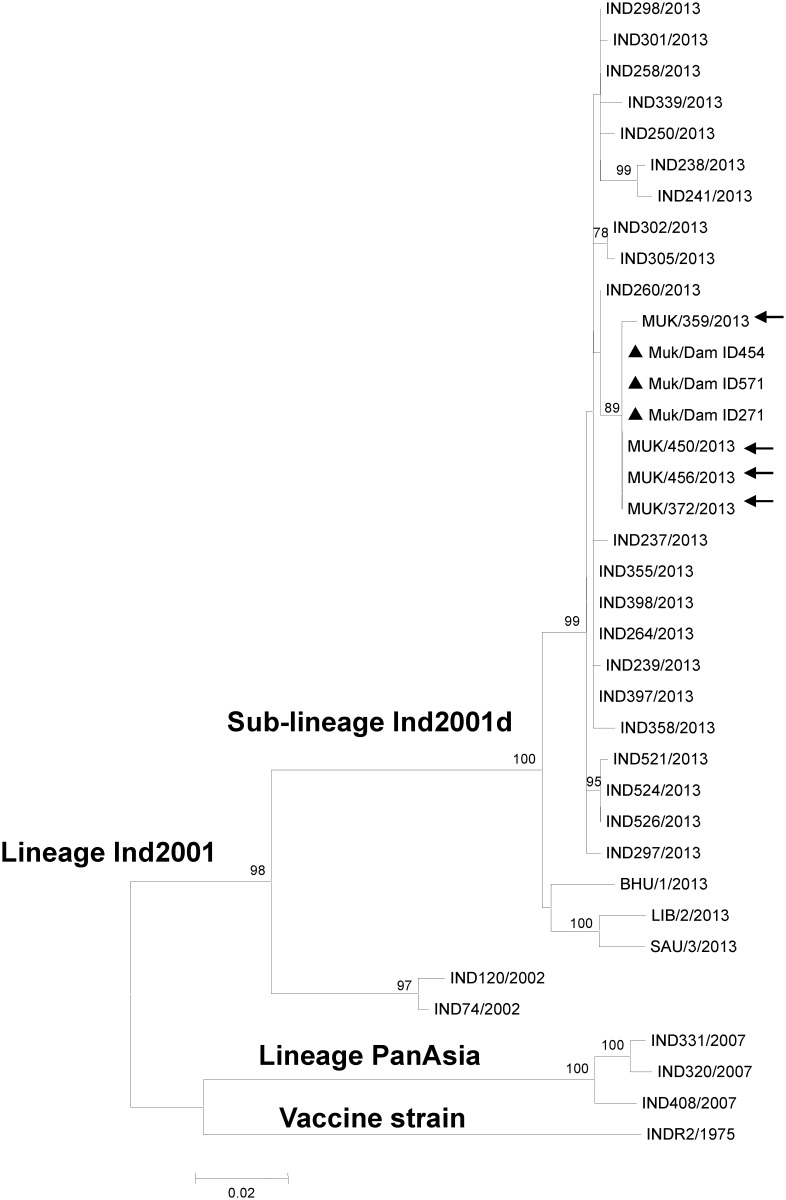
Maximum Likelihood tree depicting phylogenetic relationship of FMDV serotype O isolates. Isolates recovered from the aborted fetuses from Mukteswar farm are marked with filled triangle. Isolates recovered from adult cattle from Mukteswar farm are marked with filled circle. Bootstrap values above 70% are shown.

FMDV genomic sequences of the VP1 coding region were obtained from three of the aborted fetuses (Dam ID 454, 571, 271) from the dairy farm in Uttarakhand (Genbank accession numbers KX228159, KX228160 and KX228161). Analysis of a maximum-likelihood phylogenetic tree including these and other recent FMDV isolates from south Asia and north Africa indicated that the three serotype O isolates clustered within the Ind2001d sub-lineage of ME-SA topotype (Fig 2). Furthermore, all three abortion isolates had 100% nucleotide sequence homology to one another and there was 99.7–100% homology with the serotype O isolates collected from tongue vesicle epithelium from adult animals maintained in the same farm during the active phase of the outbreak in October 2013.

### Immunomicroscopy of tissues from aborted calves

**Fig 3:**
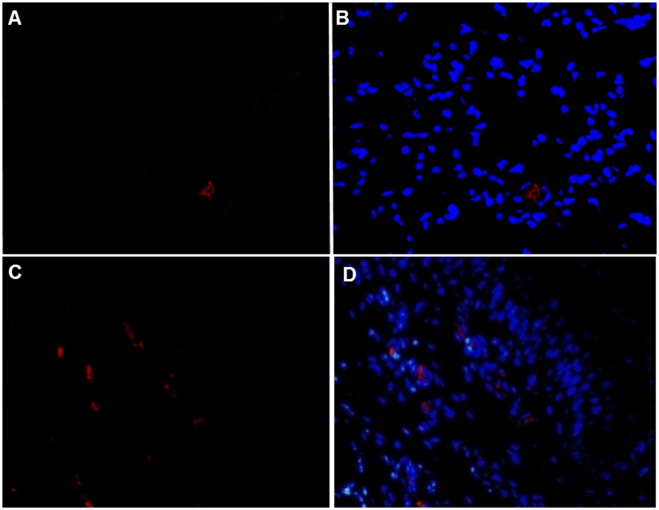
FMDV antigen detection by multi-channel immunofluorescence technique. **(A)** FMDV antigen (red) in few cells in lungs of aborted fetus from Dam ID#454 by indirect fluorescence assay (IFA). **(B)** Same image as shown in panel (A) merged with nuclear staining by DAPI (blue). **(C)** FMDV antigen (red) subjacent to basal epithelial cells in dorsal soft palate of aborted fetus. **(D)** Same image shown in panel (C) merged with nuclear staining by DAPI (blue). Rabbit anti-FMDV serotype O and rodamine-conjugated goat anti-rabbit. 40x magnification.

By use of immunomicroscopy (IHC and IFA), FMDV antigen was detected in a total of 5 tissues from two different animals (Table 2). The abortus of cows ID-454 and ID-571 had FMDV antigen in soft palate and lung (Fig 3) whereas antigen was also present in the liver of 571 (not shown). In lungs, FMDV antigen was localized to few large polygonal cells within alveolar spaces. These cells had morphology consistent with alveolar macrophages or degenerate epithelial cells (Fig 3A and 3B). In the soft palate, viral antigen was detected within the superficial subepithelium in rare individual cells or small clusters that had a perivascular distribution around small venules or capillaries (Fig 3C and 3D).

## Discussion

The work described herein represents an opportunistic field investigation of abortions amongst dairy cattle in India subsequent to an outbreak of FMD. In order to assess the causality of abortion and potential of vertical/transplacental transmission of FMDV from infected cows to fetuses, eight aborted calves from affected farms were selected and each was screened by post mortem examination. Although recent works have elucidated several aspects of FMDV pathogenesis in adult cattle [14, 15, 45–47], there are no reports regarding the pathological lesions or presence of viral genome in fetal tissues aborted from FMDV-infected cows. Additionally, there is no previous documentation of FMDV inducing abortion in cattle under experimental or field conditions.

The data herein provide strong evidence for transplacental transmission of FMDV to fetal calves and subsequent FMDV-induced abortion. Due to the unavoidable confounding factors within this field-based study, causality of the abortions was not definitely determined. Other potential causes of abortion were not ruled out as primary or contributory factors. However, recovery of FMDV RNA from fetal tissues of 7 of the 8 abortuses examined supports the premise that the calves were infected transplacentally in utero and that FMDV was the cause of abortion. The finding that 9 out of 10 tissues contained viral RNA at the earliest time point after clinical disease, but subsequent time points had lower prevalence of detection suggests a hematogenous process with subsequent decay of virus loads. These findings are consistent with previous work that demonstrated that transplacental FMDV infection in sheep occurred at 2–4 dpi (days post infection) with viral RNA detected at high levels in several tissues throughout the fetus after 7 dpi [34].

Phylogenetic analysis of FMDV strains recovered from three aborted fetuses from the Uttarakhand herd provided further support for vertical transmission from cows within the same herd. The high sequence homology (>99.7%) of the VP1 protein across outbreak and abortus-derived viruses from the same site confirmed that the strains associated with the abortions were the same strains that had recently caused clinical FMD in that location [17]. However, this finding does not definitively prove vertical transmission since outbreak viruses were not sequenced from the same cows from which abortuses were collected.

In the current study, the soft palate (7/8 cases) and palatine tonsil (5/8 cases) were the most consistent sites of detection of viral RNA suggesting that virus-host interaction in these calves favored replication at these sites. Previous studies have demonstrated that the dorsal soft palate and the dorsal nasopharynx (the floor and ceiling of the nasopharynx, respectively) is a site of unique FMDV-host tropism in adult cattle during the early [14, 15, 48] and persistent [46, 47, 49, 50] stages of FMD pathogenesis. Earlier works have demonstrated detection of viral RNA for up to two days after cessation of viremia in numerous tissues, but substantially longer duration of detection (up to 72 dpi) in pharyngeal tissues and lymph nodes [47, 51]. In the calves described herein, viral RNA was also detected in the submandibular lymph node (2/8 cases) which is consistent with previous work demonstrating FMDV antigens in lymph nodes of cattle during persistent infection [52].

Our tissue-specific data demonstrated correlation between the detection of rescued FMDV by chemical transfection and of viral RNA by GD methods. This finding suggests that the tissues from which FMDV could be rescued were the sites of the greatest quantity of viral RNA. The finding that FMDV could only be rescued from samples of soft palate further supports the role of this tissue in FMD in these calves.

We have reported herein that under the current naturally occurring conditions of infection with FMDV serotype O, gross lesions in aborted calf fetuses were limited to myocarditis and bicavitary effusions suggestive of congestive heart failure. Examination of FMDV-RNA-positive hearts from infected fetuses indicated that infection was associated with mild to moderate degeneration and necrosis of the cardiomyocytes consistent with earlier findings in sheep, goats, pigs, and adult cattle infected with FMDV [4, 34, 53, 54]. Additionally, affected hearts had variable, moderate infiltration of inflammatory cells, predominantly lymphocytes dissecting myofibers consistent with findings previously described in sheep [10], pigs [53] and cattle [55]. In previous works, FMDV-infected lambs had lymphocytic myocarditis which was considered as a significant stimulus of the cardiac myocytes to release inducible nitric oxide synthase enzyme (INOS) and thereby increase production of nitric oxide (NO) [56, 57]. Death of aborted fetuses, stillbirth and deaths of calves has been attributed to myocardial degeneration and disruption of the cardiac conduction system [58] as has been observed during post-mortem examination of affected cases. However the current report is the first instance in which FMDV-associated abortion in cattle has been associated with detection of FMDV RNA in grossly identifiable myocarditic foci.

FMDV antigens were localized by indirect immunofluorescent microscopy to only two tissue types, lungs and soft palate. This is consistent with previous works which have localized FMDV to these tissues in adult cows using immunomicroscopy [14, 15, 47] or in situ hybridization [50, 59–61]. The current localization of FMDV to subepithelial regions of the soft palate differs from previous descriptions in adult cattle. Several works have contributed to the growing understanding of the unique tropism of FMDV for the bovine nasopharynx during the acute [14, 15, 48] and persistent phase [46, 47, 49, 50] of infection. Microscopic localization of FMDV antigen has been demonstrated within the superficial epithelium of the nasopharynx of cattle [14, 15, 46, 47]. Similarly, FMDV RNA has been localized in the basal layers of the epithelium of the dorsal soft palate and pharynx [50, 61]. However, the subepithelial, perivascular localization of FMDV in the calves described herein has not been described previously. Further studies are required to describe this microanatomic localization more thoroughly. Neither histogenesis nor pathogenesis of these FMDV-containing cells was definitively determined in the work herein, but it is possible that these cells are antigen-presenting cells that are in transit between the palatal epithelium and microsvasculature. Additionally, the localization of FMDV antigens to unattached cells in pulmonary alveoli is consistent with previous conclusions that such cells were either alveolar macrophages [59] or degenerate pneumocytes [14].

## Conclusions

Detection of FMDV RNA and antigens has been demonstrated for the first time from tissues collected from aborted bovine fetuses in association with a naturally occurring outbreak of FMD. This was accomplished with viral genome detection, immunohistochemistry, and recovery of infectious FMDV through transfection. Affected tissues included three anatomic sites (hearts, lungs, and soft palate) for which FMDV is known to have selective tropism in adult cattle. These data suggest vertical transmission of FMDV from dams to fetuses during the acute phase of infection at different stages of pregnancy in cattle. The findings of this preliminary study suggest that further detailed experimental and field investigations may further elucidate the pathogenesis of *in utero* transplacental FMDV infection. It remains unresolved whether the findings described herein are specific to the FMDV-Ind2001d lineage. Additionally, further work is required to investigate whether live-born calves may become asymptomatically infected subsequent to infection *in utero* which would have substantial implications for FMDV epidemiology and control in endemic regions.
